# Debridement of Bacterial Biofilms with TiO_2_/H_2_O_2_ Solutions and Visible Light Irradiation

**DOI:** 10.1155/2018/5361632

**Published:** 2018-07-02

**Authors:** Oscar Janson, Maria Strømme, Håkan Engqvist, Ken Welch

**Affiliations:** ^1^Division of Applied Material Science, Department of Engineering Sciences, Uppsala University, Uppsala 751 21, Sweden; ^2^Division of Nanotechnology and Functional Materials, Department of Engineering Sciences, Uppsala University, Uppsala 751 21, Sweden

## Abstract

**Objectives:**

The aim of the study was to explore the debridement efficacy of different solutions of H_2_O_2_ and rutile particles against* Staphylococcus epidermidis* and* Pseudomonas aeruginosa* biofilms attached to titanium surfaces when exposed to visible light.

**Materials and Methods:**

Titanium discs cultivated with biofilms of* Staphylococcus epidermidis* or* Pseudomonas aeruginosa* were subjected for 1 min to suspensions consisting of rutile particles mixed with high (950 mM) or low (2 mM) concentrations of H_2_O_2_ under visible light irradiation (405 nm; 2.1 mW/cm^2^). Discs were rinsed and the degree of debridement was determined through scanning electron microscopy and viability assessment of the remaining bacteria using luminescence measurements and/or a metabolic activity assay.

**Results:**

Cleaning mixtures containing the higher concentration of H_2_O_2_ showed a significantly improved debridement compared to the negative control in all experiments. The addition of rutile particles was shown to have a statistically significant effect in one test with* S. epidermidis*. Limited evidence of the catalytic effect of visible light irradiation was seen, but effects were relatively small and statistically insignificant.

**Conclusions:**

H_2_O_2_ at a concentration of 950 mM proved to be the strongest contribution to the debridement and bactericidal effect of the cleaning techniques tested in this study.

## 1. Introduction

Peri-implantitis is an inflammatory disease that is defined by bleeding on probing and marginal loss of bone at the implant site [1]. The frequency of peri-implantitis has been reported to be as high as 19-22% of subjects and 9-10% of implants [2–4]. Peri-implantitis arises from bacteria attaching to the dental implants leading to the formation of bacterial biofilms, which are clusters of bacteria encased in an extracellular polymeric substance and adherent to a surface [5]. The bacteria in biofilms possess a different gene expression compared to planktonic bacteria which can make the infection resistant to antibiotics [6]. Therefore nonantibiotic treatment alternatives may be required. Commonly used cleaning protocols for peri-implantitis infected implants include mechanical cleaning with curettes or brushes, which can damage the implant surface and have difficulty reaching bacteria located in the micro roughness of the implants [7]. Chemicals like hydrogen peroxide (H_2_O_2_), chlorhexidine gluconate, saline, and citric acid are also used for disinfection and while they all show good effect, they are not satisfactory for complete cleaning of the implants [8]. Therefore there is an urgent need for more effective cleaning methods.

Photocatalytic activation of titanium dioxide (TiO_2_) has long been investigated not only as an environmental cleaning agent of waste water and air purification [9], but also for biomedical applications [10–12]. Photocatalysis occurs when an electron from the valence band in a TiO_2_ crystal is excited to the conduction band by a photon of higher energy compared to the band gap between the valence and conduction bands of the crystal, thereby creating an electron-hole pair. The band gaps for the two most common crystal forms of TiO_2_, anatase and rutile, are 3.20 and 3.03 eV, respectively [13], corresponding to light wavelengths of 387 nm and 409 nm, respectively. The excited electron can react with oxygen (O_2_) and create superoxide radicals (O_2_^−∙^) while the hole can react with water (H_2_O) and give rise to hydroxyl radicals (^*∙*^OH) [14]. Reactive oxygen species (ROS) such as hydroxyl radicals, superoxide radicals, and H_2_O_2_ are known to damage the cell wall of bacteria leading to cell death [15]. Rutile has a smaller band gap but is considered a less active photocatalyst compared to anatase or mixed anatase/rutile phases [16]. However when adding small amounts of H_2_O_2_, rutile has been shown to increase hydroxyl radical formation under light irradiation [16–18]. H_2_O_2_ is in itself an effective antiseptic and with a concentration of 3 vol.% (950 mM) has been shown to be an effective treatment against* S. epidermidis* biofilms on titanium dental implant surfaces* in vitro* [19–21]. TiO_2_ in combination with H_2_O_2_ has been shown to give rise to an increased synergistic bactericidal effect [19, 21]. Studies have shown that small amounts of H_2_O_2_ together with TiO_2_ of the allotropic form rutile under light exposure is the most effective combination to degrade the organic dye Rhodamine B [22], as well as inactivate the multiresistant bacterium* Klebsiella pneumoniae* [23].

We hypothesize that TiO_2_ particles combined with H_2_O_2_ under light irradiation can be effective in debriding peri-implantitis infected implants. Consequently, the aim of this study was to explore the debridement efficacy of mixtures of rutile TiO_2_ particles and H_2_O_2_ under visible light irradiation (405 nm wavelength) on* Staphylococcus epidermidis* and* Pseudomonas aeruginosa *biofilm adherent to Ti surfaces.* S. epidermidis* is a Gram-positive bacterium that easily can create biofilms [15] and* P*.* aeruginosa* is a Gram-negative, rod-shaped bacterium which has a low antibiotic susceptibility, making this pair suitable for the test in the current study. Both species are facultative anaerobes, which means that they can grow both with and without oxygen.

## 2. Material and Methods

### 2.1. Titanium Discs

Commercially pure (cp), machine shaped Ti discs (diameter 6.2 mm, thickness 2 mm) were surface-modified to mimic a rough dental implant surface. The discs were grit blasted with TiO_2_ particles (particle size = 180-220 *μ*m) and further acid etched with 0.2 vol.% hydrofluoric acid. A detailed description of the modification procedure and a detailed characterization of the surface can be found elsewhere [16].

### 2.2. Bacteria Strains and Biofilm Formation

Two bacteria strains were selected to form biofilms in this study. First, a genetically modified Gram-positive strain of* S. epidermidis* XEN 43 was used. The strain was derived from the clinical isolate* S. epidermidis* 1457, where luxABCDE genes have been incorporated into the bacterial genome to create a constitutive bioluminescent strain. The engineered strain has been shown to be phenotypically equal to its parental strain [17] and was stored as frozen stock cultures at −20°C in 15% glycerol (American Bioanalytic, Natick, MA, USA) to avoid phenotypic changes due to passages. Tryptic soy broth (TSB) with 10 % glucose syrup (Dan Sukker, Nordic Sugar AB, Malmö, Sweden) was used as growth medium and incubation was performed in an aerobic atmosphere at 37°C. Biofilm inoculum for Ti discs was prepared by adding 10 *μ*l stock to 10 ml growth medium and inoculating overnight before adjusting the OD at 600 nm to 0.02 (~ 1 x 10^7^ cells ml^−1^). 600 *μ*l of the bacterial suspension was added to Ti discs placed in a 48-well microtiter plate (Nunc, Roskilde, Denmark) and then incubated for 24 h to allow colonization and biofilm formation on the discs. Details on determination of the correlation between luminescence and colony forming units (CFU), as well as OD_600_ and CFU, can be found elsewhere [18].

The second bacteria strain used was the Gram-negative, rod-formed bacterial species* P. aeruginosa* ATCC 39324, which is a mucoid clinical isolate from a cystic fibrosis patient. 200 *μ*l of precultures of* P. aeruginosa *was added to 10 ml Tryptic soy broth (TSB) with 0.2 % (v/v) of glycerol and inoculated in aerobic conditions in 37°C overnight. Ti discs were then inoculated with 600 *μ*l of bacterial suspension diluted to OD_600_ = 0.2 and then incubated for 24 h to allow colonization and biofilm formation on the discs.

### 2.3. Cleaning (Debridement) Procedure

Table 1 details the composition of the sample cleaning mixtures and the treatment conditions. TiO_2_ particles used were of the allotropic form rutile. As a positive control 70% ethanol was used, prepared from mixing 96% ethanol (VWR, Fontenay-sous-Bois, France) with deionized water. Dulbecco's phosphate buffered saline with added Mg^2+^ and Ca^2+^ (PBS) was used as negative control and also as solvent in cleaning mixtures. All chemicals including the rutile particles were purchased from Sigma Aldrich (Sigma Aldrich, St Louis, MO, USA) if not stated differently.

After the biofilm formation procedure detailed in Section 2.2, discs were moved to another 48-well microtiter plate containing a solution with sterile PBS. Before debridement discs with biofilm were washed three times in PBS to remove bacteria that were not attached to a biofilm. This washing procedure was not used within a second set of tests with* S. epidermidis* discs as it was found that the biofilm had too poor adherence to the surface. From there the discs were transferred to new wells and 200 *μ*l of the chemical agents listed in Table 1 was added. Mixtures containing TiO_2_ particles were vortexed immediately before use. Treatment was stopped after 1 min by transferring the discs to separate wells filled with 1 ml PBS. Discs were then rinsed by pipetting sterile PBS three times to remove all chemical agents before further processing. All test groups consisted of 9 replicates from which 1 was used for scanning electron microscopy (SEM) assessment.

### 2.4. Assessment of Debridement and Bacterial Viability

#### 2.4.1. Scanning Electron Microscopy

One disc from each test group was removed for examination with SEM. The discs were fixated in 2.5% glutaraldehyde for 1 h, followed by immersion in ethanol (30, 50, 70, and 96% ethanol) for 10 min in each concentration. Subsequently the discs were soaked in hexamethyldisilane (HMDS) for 15 min and then left to dry for a couple of hours. Afterwards the discs were sputter-coated with a thin Au/Pd layer for increased signal and reduced charging effects. The discs were then imaged using the secondary electron detector in a Leo 1530 scanning electron microscope (Zeiss, Oberkochen, Germany) operated at 5.0 kV.

#### 2.4.2. Metabolic Activity Assay (MAA)

The postdebridement viability of both bacterial strains was analyzed using the MAA. 400 *μ*l of MAA solution composed of the metabolic indicator resazurin at a concentration of 12.5 *μ*g/ml in TSB was added to each well containing a sample disc. Fluorescence measurements were made every hour for 6 hours in a Tecan M200 fluorescence spectrometer, with excitation wavelength set at 530 nm and emission wavelength at 590 nm. Between measurements the plates were placed on a shaker board in 37°C. Wells containing only TSB were also measured to determine the background fluorescence, which was then subtracted from all test groups.

#### 2.4.3. Luminescence Measurements

Regrowth of* S. epidermidis* bacteria was also measured by monitoring luminescence after a separate debridement test. In this test, washing of the discs before the debridement procedure was not performed as it was observed in the previous MAA experiment that prewashing removed too much of the biofilm. After the debridement procedure, eight discs of each group were transferred to a new 48-well plate and the wells were filled with 600 *μ*l of TSB. Luminescence was recorded at the start of regrowth (0 h) and once every hour from 8 h to 18 h after start of regrowth in a Hidex chameleon plate reader. Between measurements the plates were stored at 37°C. A well containing a sterile Ti disc was also measured to ensure that the growth media or Ti substrate did not contribute to the luminescent signal. For comparison of regrowth between sample groups, the time for samples to reach 2000 luminescence units was used.

### 2.5. Statistical Analysis

The results are presented as the mean ± standard error of the mean. Statistically significant differences from the mean were calculated with one-way ANOVA, followed by Dunnett and Tukey post hoc tests. The Dunnett post hoc test was used to compare each test group against the PBS control while the Tukey post hoc test was used to confirm statistically significant differences amongst the four test groups and positive control. The Anderson-Darling test was used to verify if the data sets were normally distributed and Levene's test was used to assess the equality of variances.

## 3. Results

### 3.1. MAA:* S. epidermidis*

**Figure 1:**
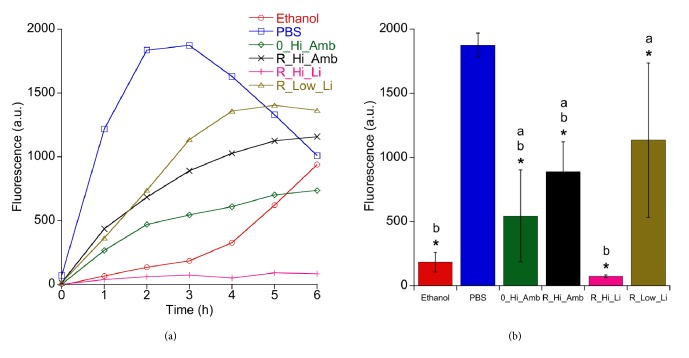
Regrowth of S*. epidermidis* after different debridement treatments. Background fluorescence from TSB was subtracted from all test groups. (a) Average fluorescence intensity measured during reincubation after debridement with different treatments (n=8). (b) Average fluorescence intensity after 3 h regrowth. Groups with an asterisk indicate statistically significant difference compared to the control PBS (Dunnett post hoc test, p≤ 0.05). Groups with different letters are significantly different (five test groups compared, Tukey post hoc test, p ≤ 0.05).

**Figure 2:**
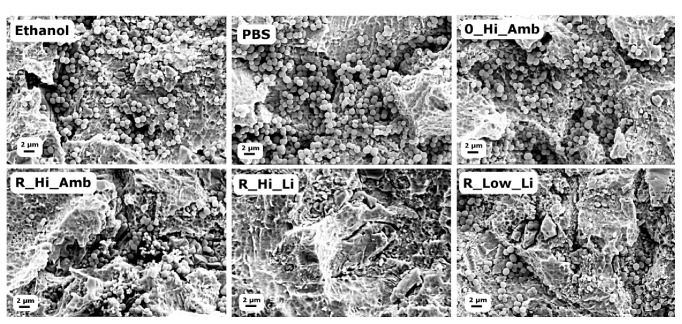
SEM micrographs of* S. epidermidis* on the Ti surfaces after the different debridement treatments.

Figure 1 shows the viability of* S. epidermidis *after the debridement treatments as measured with the MAA. All five test groups exhibited statistically significantly lower fluorescence intensities after 3 h regrowth compared to the PBS treated Ti discs (Figure 1(b)). The positive control discs treated with ethanol showed a strong bactericidal effect in the MAA, but after three-hour reincubation, bacterial growth increased at a higher rate compared to the other groups; cf.  Figure 1(a). It can also be seen in the SEM micrographs in Figure 2 that the ethanol treatment did not effectively debride the biofilm as a relatively large amount of bacteria remained on the surface compared to the discs treated with PBS. On the other hand, the R_Hi_Li treatment appeared to have produced the most efficient debridement as very few bacterial cells could be observed on the disc surface, which was also supported by the lowest fluorescence values recorded in the MAA. However, it should be noted that due to the relatively poor adhesion of the* S. epidermidis *biofilm to the discs, a large percentage of the bacteria was removed from all discs during the washing procedure before debridement, which likely contributed to the high variability in the recorded data. Consequently, the lower viability of R_Hi_Li compared to 0_Hi_Amb and R_Hi_Amb in Figure 1(b) was not statistically significant (Tukey post hoc test, p≤ 0.05).

### 3.2. Luminescence Assay:* S. epidermidis*

**Figure 3:**
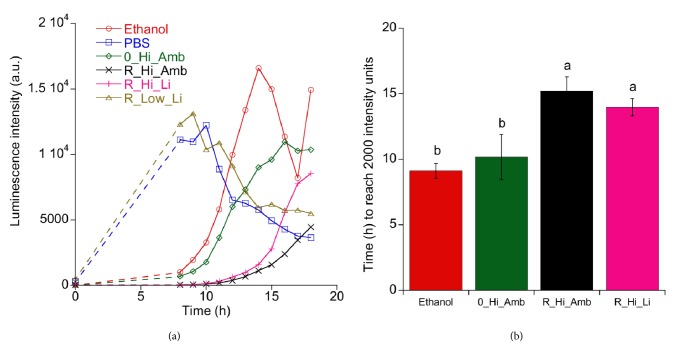
Regrowth of* S. epidermidis* after different debridement treatments. (a) Average luminescence intensity measured during reincubation after debridement with different treatments (n=8). (b) Average time after reincubation to reach a luminescence intensity unit of 2000 for four of the test groups. Different letters associated with the bars indicate statistically significant differences (Tukey post hoc test, p ≤ 0.05).

Figure 3 displays the viability of* S. epidermidis* postdebridement treatment, as measured with the luminescence assay. As can be seen in panel (a), the negative control (i.e., PBS treatment) and R_Low_Li samples had the highest viability and both showed a peak in the regrowth curve at approximately 8 h (note that luminescence measurements were not made between the initial measurement at 0 h, just after the cleaning treatments, and 8 h). The positive control (ethanol) had an earlier/faster regrowth than the test groups containing higher concentrations of H_2_O_2_, which may be expected since the previous MAA with* S. epidermidis *showed that the activity or growth of bacteria on the ethanol discs increased at an accelerated rate after three hours of reincubation. Because luminescence measurements were not taken between the initial measurement at 0 h and 8 h, it is not possible to assess the viability of the ethanol discs compared with the other sample groups in this period.

**Figure 4:**
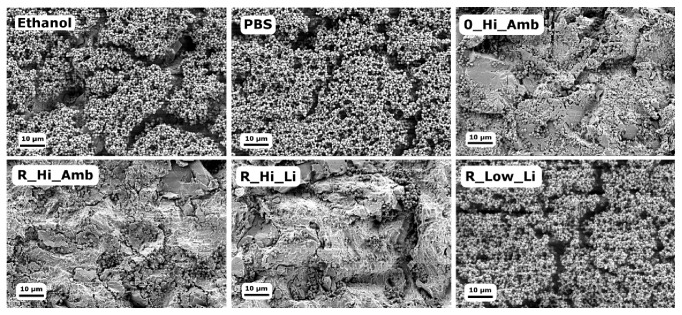
SEM micrographs of* S. epidermidis* on the Ti surfaces after the different debridement treatments.

Similar to the previous MAA, Figure 4 shows that a relatively large amount of bacteria remained on the ethanol discs, suggesting that while ethanol treatment kills or inactivates the bacteria, it does not debride the biofilm. Note that the washing procedure before debridement was not performed in this test, which is why, relatively, so many more bacteria are observed in Figure 4 compared to Figure 2.

Figure 4 also shows that the debridement treatment with high H_2_O_2_ concentrations resulted in efficient debridement of the* S. epidermidis *biofilm, which is supported by luminescence measurements. The addition of TiO_2_ particles to the H_2_O_2_ solutions yielded a significant delay in regrowth time; cf.  Figure 3(b). However, irradiation with 405 nm light did not seem to have additional benefits as was suggested by the trend in the MAA measurements.

### 3.3. MAA:* P. aeruginosa*

**Figure 5:**
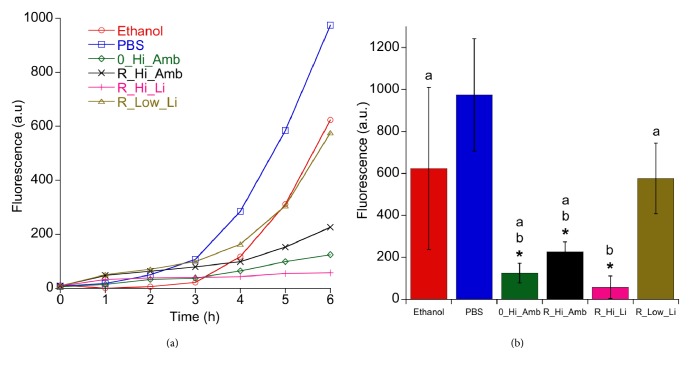
Regrowth of* P. aeruginosa* after different debridement treatments. (a) Fluorescence measured during reincubation after debridement with different treatments (n=8). (b) Average fluorescence intensity after 6 h regrowth. Groups with an asterisk indicate statistically significant difference compared to the control PBS (Dunnett post hoc test, p≤ 0.05). Groups with different letters are significantly different (five test groups compared, Tukey post hoc test, p ≤ 0.05).

**Figure 6:**
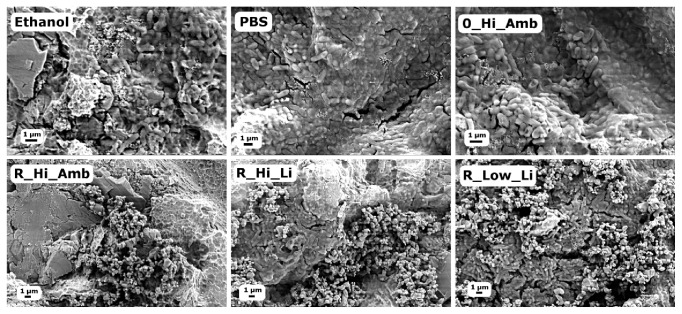
SEM micrographs of* P. aeruginosa* on the Ti surfaces after the different debridement treatments.

Figure 5 displays the viability of* P. aeruginosa* after the debridement treatments as measured with the MAA. The Ti discs treated with debridement solutions containing the higher concentration of H_2_O_2_ all showed a significantly lower fluorescence signal at six hours after debridement compared to the Ti discs treated with only PBS; see Figure 5(b). An assessment time of 6 h was chosen instead of 3 h as was used with* S. epidermidis *due to the relatively slower growth rate of the* P. aeruginosa *bacteria. It is notable that the positive control ethanol did not show a significantly lower signal than PBS at 6 h, but this is likely due to an increased activity or growth rate after an initial period of reincubation as was seen with* S. epidermidis. *Inspection of the viability curves in Figure 5(a) gives the fact that the viability of the ethanol group is in fact the lowest for the first three hours of reincubation.  Figure 6 displays SEM micrographs of the Ti surfaces after the debridement treatments, which show a very different morphology of the* P. aeruginosa* biofilm compared with the* S. epidermidis* biofilm.  Figure 6 as well shows that a substantial amount of rutile particles is left after the debridement treatment.

## 4. Discussion

In this study the antibacterial effect and debridement efficacy of mixtures of H_2_O_2_ and TiO_2_ particles with and without visible light irradiation was performed on* S. epidermidis* and* P. aeruginosa* biofilms grown on Ti surfaces. The choice of the Gram-positive* S. epidermidis* was made because of its good biofilm forming abilities and this special strain Xen 43 provides the ability to monitor viability using luminescence measurements, giving a direct indication of bacterial proliferation [21, 24].* P. aeruginosa *was chosen because it is a Gram-negative strain, which generally has a stronger resistance against antimicrobials [25]. The higher H_2_O_2_ concentration of 950 mM (3 vol.%) was chosen because it is used clinically today to debride peri-implantitis infected implants while the lower concentration of 2 mM (0.006 vol.%) was chosen because it has been shown to be the optimal concentration to degrade an organic substance when combined with rutile particles under visible light irradiation [22].

It was observed that the debridement mixtures with high concentrations of H_2_O_2_ (950 mM) produced a large bubbling effect when applied on the biofilm-covered surface. The high reduction of biofilm associated with high concentrations of H_2_O_2_ seen in the* S. epidermidis *test assessed with luminescence may thus be correlated to the oxygen evolution evidenced by the bubbles that are formed [19–21]. These bubbles are caused by H_2_O_2_ being decomposed by catalase produced by the bacteria. The bubbles in turn may create mechanical forces in the solution that remove the attached bacteria. This effect was most apparent in the luminescence test with* S. epidermidis *because more bacteria were present on the surface prior to the debridement treatments compared to the MAA test with* S. epidermidis.* The first debridement test using* S. epidermidis* showed that the bacteria were poorly attached to the Ti surface since a large proportion was removed with just a gentle rinsing with PBS prior to the debridement treatment, and thus this prewash was not performed with the luminescence test. With the addition of rutile particles there was an increased bactericidal effect when measured with luminescence, possibly because the particles added an extra mechanical force to the bubbling effect, which is in line with several other studies [19, 21]. However, it could also be due to a catalytic effect of the rutile particles on H_2_O_2_ to generate additional ROS [19, 21].

The addition of light irradiation at a wavelength of 405 nm did not provide a statistically significant extra effect, although a trend for increased effect was seen in the MAA tests with both bacteria species when combined with a high H_2_O_2_ concentration. Theoretically this wavelength has enough energy to induce photocatalysis in the rutile particles used in this study, which could produce additional ROS. The lack of a clear effect may be because hydroxyl radicals, which are the main bactericidal ROS created in photocatalysis, are very reactive and consequently would have a reaction distance in the magnitude of micrometers [10, 21]. This would limit the effect to the immediate surroundings of the particles, which may not be close enough to the majority of the bacteria based on the particle concentrations used in this study. In the luminescence experiments, no effect of irradiation was seen, but this may partially be due to the fact that the mechanical debridement from the evolved oxygen bubbles removed the majority of the biofilm and with it the overlaying rutile particles.

Ethanol had a stronger effect on* S. epidermidis* in the MAA experiment compared to* P. aeruginosa. *The smaller effect against* P. aeruginosa* is expected since this strain is more resistant against ethanol compared to* S. epidermidis* [26]. In all tests, biofilms treated with ethanol showed an increased activity or regrowth after a delay period during reincubation. This highlights the advantages of the other debridement treatments used in this study.

In summary, H_2_O_2_ at a concentration of 950 mM proved to be the strongest contribution to the debridement and bactericidal effect of the cleaning techniques tested in this study. The addition of rutile particles may have additional benefit and was shown to have a statistically significant effect in one test with* S. epidermidis*. Limited evidence of the catalytic effect of visible light irradiation was seen, but effects were relatively small and statistically insignificant. Further studies with clinically relevant and multispecies biofilm should be performed to confirm these results. The debridement of dental implants with H_2_O_2_ and rutile particles under visible light irradiation can be used as an alternative or complementary technique to presently used methods such as mechanical debridement or chemical disinfection in the treatment of peri-implantitis.

## Figures and Tables

**Table 1:** Description of debridement treatment conditions. Exposure time was 1 min for all treatment conditions.

Sample designation	Concentration rutile (g/l)	Concentration H_2_O_2_ (mM)	Light, intensity:wavelength (mW/cm^2^:nm)
Ethanol (Positive control)	-	-	Ambient light
PBS (Negative control)	-	-	Ambient light
0_Hi_Amb	-	950	Ambient light
R_Hi_Amb	0.5	950	Ambient light
R_Hi_Li	0.5	950	2.1:405
R_Low_Li	0.5	2	2.1:405

## Data Availability

The data used to support the findings of this study are included within the article.
